# Single-Cell RNA Sequencing Unravels Heterogeneity of the Stromal Niche in Cutaneous Melanoma Heterogeneous Spheroids

**DOI:** 10.3390/cancers12113324

**Published:** 2020-11-10

**Authors:** Jiří Novotný, Karolína Strnadová, Barbora Dvořánková, Šárka Kocourková, Radek Jakša, Pavel Dundr, Václav Pačes, Karel Smetana, Michal Kolář, Lukáš Lacina

**Affiliations:** 1 Laboratory of Genomics and Bioinformatics, Institute of Molecular Genetics of the Czech Academy of Sciences, 142 20 Prague, Czech Republic; jiri.novotny@img.cas.cz (J.N.); sarka.kocourkova@img.cas.cz (Š.K.); vaclav.paces@img.cas.cz (V.P.); 2 Department of Informatics and Chemistry, Faculty of Chemical Technology, University of Chemistry and Technology, 160 00 Prague, Czech Republic; 3 Institute of Anatomy, First Faculty of Medicine, Charles University, 128 00 Prague, Czech Republic; karolina.strnadova@lf1.cuni.cz (K.S.); barbora.dvorankova@lf1.cuni.cz (B.D.); karel.smetana@lf1.cuni.cz (K.S.J.); 4 BIOCEV, First Faculty of Medicine, Charles University, 25250 Vestec, Czech Republic; 5 Institute of Pathology, First Faculty of Medicine, Charles University, 128 00 Prague, Czech Republic; radek.jaksa@vfn.cz (R.J.); pavel.dundr@lf1.cuni.cz (P.D.); 6 Department of Dermatovenereology, First Faculty of Medicine, Charles University and General University Hospital, 128 00 Prague, Czech Republic

**Keywords:** melanoma, spheroids, fibroblasts, subpopulation, heterogeneity, Interleukin-6, cytokine, senescence-associated secretory phenotype, extracellular matrix, single-cell sequencing

## Abstract

**Simple Summary:**

Cutaneous malignant melanoma is one of the most dangerous forms of skin cancer affecting humans. Frequently, it is linked to DNA damage due to ultraviolet radiation. Photoageing along with chronological ageing are therefore critically important factors in melanoma biology. The tissue microenvironment is also heavily affected by induced senescence. There is growing evidence that senescent dermal fibroblasts can consequently promote tumour progression. We focused on the analysis of microenvironmental factors represented in melanoma heterogeneous spheroids by either photodamaged or normal dermal fibroblasts. Our effort was primarily focused on the determination of the functional diversity of fibroblasts in heterogeneous spheroids. Therefore, we analysed these 3D models by single-cell RNA sequencing and advanced bioinformatic analysis. We aimed to map the fibroblast diversity resulting from previously acquired damage caused by exposure to extrinsic and intrinsic stimuli. Using this robust methodology, we highlighted molecules that could become important for the control of melanoma cell–cancer-associated fibroblast interaction as an essential part of the tumour microenvironment.

**Abstract:**

Heterogeneous spheroids have recently acquired a prominent position in melanoma research because they incorporate microenvironmental cues relevant for melanoma. In this study, we focused on the analysis of microenvironmental factors introduced in melanoma heterogeneous spheroids by different dermal fibroblasts. We aimed to map the fibroblast diversity resulting from previously acquired damage caused by exposure to extrinsic and intrinsic stimuli. To construct heterogeneous melanoma spheroids, we used normal dermal fibroblasts from the sun-protected skin of a juvenile donor. We compared them to the fibroblasts from the sun-exposed photodamaged skin of an adult donor. Further, we analysed the spheroids by single-cell RNA sequencing. To validate transcriptional data, we also compared the immunohistochemical analysis of heterogeneous spheroids to melanoma biopsies. We have distinguished three functional clusters in primary human fibroblasts from melanoma spheroids. These clusters differed in the expression of (a) extracellular matrix-related genes, (b) pro-inflammatory factors, and (c) TGFβ signalling superfamily. We observed a broader deregulation of gene transcription in previously photodamaged cells. We have confirmed that pro-inflammatory cytokine IL-6 significantly enhances melanoma invasion to the extracellular matrix in our model. This supports the opinion that the aspects of ageing are essential for reliable melanoma 3D modelling in vitro.

## 1. Introduction

Cutaneous malignant melanoma is one of the most dangerous forms of skin cancer in humans. This life-threatening solid tumour can be linked to the DNA damage due to ultraviolet radiation from natural and artificial sources [1]. Epidemiological studies have identified that intermittent sunburns leading to inflammation constitute a significant risk factor in the development of malignant cutaneous diseases. The sunburn injury depends on the absorbed UV dose, UV wavelength applied, and finally also on the skin phototype. Similar to other types of tumours, the melanoma incidence is increasing with age because of the reduction of gene repair machinery in post-reproductive life [2,3,4]. This highlights the importance of chronological ageing in cancer research.

The formation of dipyrimidine photoproducts by direct absorption of UV energy in DNA can lead to the so-called UV signature mutations [5]. UV exposure also causes generation of reactive species through various pathways. Oxidative DNA damage may also, to some extent, play a role in photocarcinogenesis [6]. Genotoxic effects of skin irradiation can combine in epidermal cells, and thus contribute to cancer initiation and progression [7]. In a deeper layer, skin photoageing is caused by the repeated exposure of the dermis predominantly to UVA radiation. There is also evidence that repeated UVA irradiation of fibroblasts alters functions in photoaged skin, and it is an essential factor of deeper cancer invasion [8].

Regardless of the mechanism of senescence induction, senescent cells do accumulate in the organism with age [2]. This may also lead to deleterious effects. Impaired cellular functions may contribute to tissue structure degeneration and functional impairment, with the consequent onset of various inflammatory disorders and many other age-associated pathologies. Surprisingly, this list would also include cancer. In contrast to what was mentioned above, this makes senescence a double-edged sword [9,10].

The definition of senescence remains primarily functional. Senescent cells can be, to a certain extent, also characterised by morphological changes, and these changes are distinct in cell cultures [11]. However, the pathological identification of individual senescent cells in complex tissues remains somewhat troublesome [12]. Although it was previously believed that fibroblasts are homogeneous and mostly quiescent cells, it has become increasingly recognised that numerous fibroblast subtypes with unique functions and morphologies exist [13]. Importantly, senescent cells broadly alter their gene expression. Therefore, single-cell transcriptome analysis seems to be of crucial importance in this research. Compared to normal juvenile cells, senescence results in a pro-inflammatory secretory phenotype occurring without any usual pro-inflammatory stimulus. It is known as the senescence-associated secretory phenotype (SASP).

There is evidence that senescent dermal fibroblasts can promote tumour progression by activating their SASP [14]. This phenotype is triggered in the dermis by either extrinsic stimuli (oxidative stress, UV-related DNA damage), or intrinsic factors of chronological ageing. Fibroblasts are consequently switched into pro-inflammatory cells, acquiring the ability to affect adjacent cells of the local microenvironment [15]. Senescent fibroblasts produce numerous factors, e.g., cytokines, chemokines, growth factors or extracellular matrix proteins [9]. Besides dermal fibroblasts and their secreted factors, the tissue microenvironment consists of the extracellular matrix, endothelial and immune cells, and the network of blood and lymphatic vessels. These elements in the deep inner skin layer collectively form a complex niche beneficial for tumour initiation and spreading. This can, e.g., drive melanoma cells to relocate from the epidermis into the dermis. The dermal fibroblasts accidentally surrounding a malignant tumour are exposed to massive intercellular signalling and alter their phenotypic profile. These initially innocent bystanders are converted into cancer-associated fibroblasts (CAFs), which are conducive to cancer growth and metastasis [16,17,18]. The cancer cells form, along with their noncancerous partners (also including CAFs and immune cells), a complex ecosystem [19,20] whose understanding is fundamental for the development of new procedures of anticancer therapy.

In earlier years, the in vitro methods of cancer research investigating, e.g., the effect of anticancer drugs were limited to the study of individual cell lines in culture dishes. This concept was somewhat misleading, because it completely neglected the important biological aspect of intercellular interactions [20,21]. Later, the crosstalk between various cells was extensively investigated, again primarily using 2D monolayer cultures that were in physical contact or separated by porous membranes [22,23,24]. To some extent, these simplistic models introduced the microenvironment. However, they were far from the complexity of real tumours, and that is why they are recently being abandoned. In the recent decade, research is focusing on 3D cultures or organotypic modelling. This helps to acquire higher complexity and brings more relevant data closer to in vivo conditions. It was exemplified on the study of apoptosis of melanoma cells in 3D culture by tumour necrosis factor α-related apoptosis-inducing ligand (TRAIL) [25]. Data from 3D cultures can more closely mimic the clinical setting, and this justifies their employment [26]. This trend was seen in melanoma and can be confirmed by data from multiple other types of tumours [27].

Employment of other cell types can further increase the complexity of the acquired model. These heterogeneous spheroids are an intermediate stage between non-complex 2D cultures and in vivo animal models [28,29,30]. The addition of dermal fibroblasts achieves the goal of making in vitro melanoma heterogeneous spheroids better resemble the melanoma tumour. Fibroblasts represent structural and metabolic support and provide the microenvironmental cues occurring in human malignant melanoma [31]. However, several critical questions must be addressed. For instance, are human dermal fibroblasts in standard cultures a homogenous population? As noted earlier, there is an unexpected natural heterogeneity of the mesenchymal populations of the dermis in the tissues [32]. Is it maintained in the cultures, and for how long? If so, should we also take ageing into account in these models [33]?

Therefore, we focused on the analysis of microenvironmental factors represented in melanoma heterogeneous spheroids by different fibroblasts. We focused on characterisation of the functional diversity of these fibroblasts in heterogeneous spheroids consisting of melanoma cells and fibroblasts. Our interest was predominantly oriented on characterisation of the functional features of dermal fibroblasts in 3D coculture with melanoma cells with respect to the fibroblast donor’s age and previous skin UV exposure. The biologically relevant variables were represented by employment of either primary human dermal fibroblasts from the sun-protected skin of a juvenile donor or fibroblasts from the sun-exposed photodamaged skin of an adult donor. The spheroids were analysed by single-cell RNA sequencing and advanced bioinformatic analysis. Using this robust methodology, we focused on molecules that could be potential therapeutic targets in the melanoma microenvironment. We used the BRAF mutated melanoma cell line. The effect of BRAF treatment was not tested and our results did not address the effect of this therapy on the fibroblast heterogeneity.

## 2. Results

### 2.1. Description of Heterogeneous Spheres Formed from the Melanoma Cells and Fibroblasts

**Figure 1:**
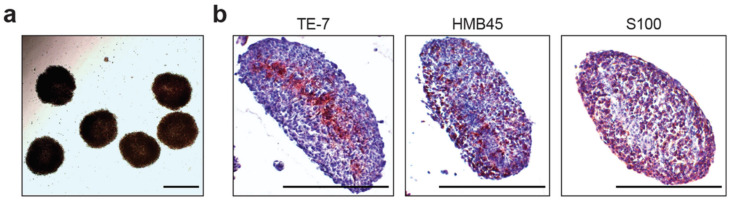
Melanoma heterogeneous spheroids (**a**) of typical regular morphology harvested for experiments. Spheroids display consistent localisation of melanoma cells on the periphery and fibroblasts in their core. The spheroids show structural inhomogeneity with fibroblasts marked by TE-7 antibody in their core and melanoma cells on their periphery visualised by HMB45 and S100 markers (**b**). The bar denotes 1000 μm.

Compact heterogeneous spheres were formed from suspensions of melanoma cells (cell line G361) and multiple primary human dermal fibroblasts (Figure 1a).

Surprisingly, we observed a uniform structural pattern in our spheres, which was revealed by immunohistochemistry (Figure 1b). Invariably, fibroblasts occupied the core of the spheres. The mantle zone of the sphere was predominantly formed by melanoma cells. We detected fibroblasts by specific TE-7 antibody. We also detected melanocyte markers such as HMB45, tyrosinase and protein S100 in melanoma. These markers were also observed in cells of the peripheral zone in spheroids (Figure 1, antibodies in Supplementary Table S1). For closer analysis by scRNA-seq, we used spheres from juvenile dermal fibroblasts (JDF, isolated from a child sun-protected skin) and adult fibroblasts from the skin, revealing clinically extensive signs of photoageing due to previous UV irradiation (PDF).

### 2.2. scRNA-seq Comparative Analysis of Fibroblasts and Melanoma Cells 

After the dissociation of spheres to single-cell suspensions, we were able to separate individual fibroblasts from G361 melanoma cells using scRNA-seq (Supplementary Figure S1). We identified fibroblasts clearly by expression of fibroblast-activating protein (*FAP*), a widely used marker of fibroblasts. Melanoma cells were identified by expression of marker MELAN-A (*MLANA*). Expression of these markers at the mRNA level could also be confirmed at the protein level by immunohistochemistry in sections of spheroids. Moreover, the relevance of these markers was also supported by findings in human samples of melanoma and its lymph node metastases from 10 patients (Supplementary Figure S1a, with detailed information regarding tumours in Supplementary Table S1).

We could further demonstrate the reliability of the scRNA-seq procedure by examples of several other typical melanoma proteins in human tumours and transcripts in spheroids (heatmap shown in Supplementary Figure S2) and also by a known CDKN2A mutation present in the G361 melanoma cell line (Supplementary Figure S3).

The genes for markers melanogenesis tyrosinase (*TYR*) and transcription factor MiTF (*MITF*), which are specific for melanoma cells, were observed predominantly in the pool of melanoma cells (Supplementary Figures S4 and S5). *CDH1* (E-cadherin) and *CDH2* (N-cadherin) were also dominant in the melanoma cell pool. This observation corresponded to the weak but specific signal from clinical samples (Supplementary Figure S6). On the other hand, cells producing the extracellular matrix (for example *COL1A2*) and expressing an active gene for a protein participating in collagen metabolism such as *LOX*, were particularly enriched in fibroblast cells (Supplementary Figures S4 and S5). As expected, mRNA transcripts for intermediate filament vimentin (*VIM*) were strongly transcribed in both cell types, i.e., in fibroblasts and melanoma cells (Supplementary Figures S4 and S5) and also in clinical samples (Supplementary Figure S6). While we observed significant differences between melanoma cells and both types of fibroblasts (heatmap in Supplementary Figure S2), we analysed these cell populations separately for further stratification.

### 2.3. Bioinformatic Analysis of Primary Dermal Fibroblasts (JDF vs PDF) Dissociated from Spheres

**Figure 2:**
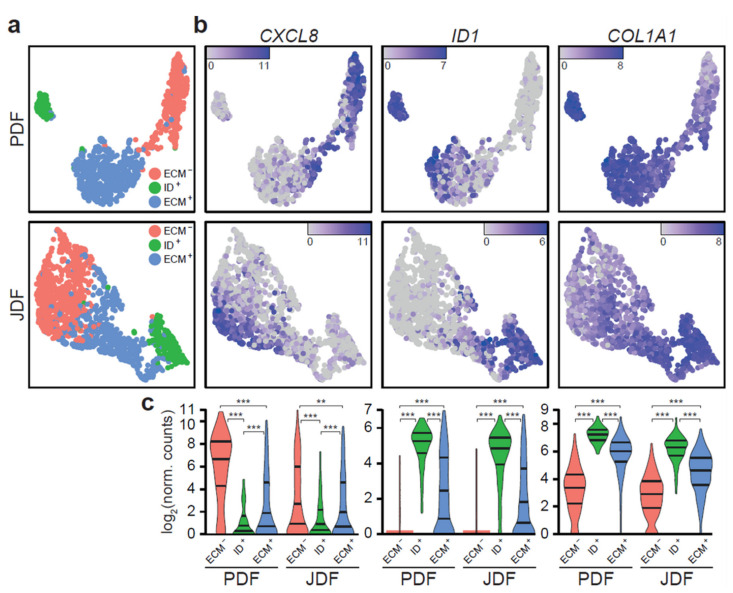
Fibroblasts in JDF and PDF spheroids split into three phenotypically distinct clusters. (**a**) The clusters are defined by specific gene expression signatures and separate well in the UMAP visualisation. (**b**) ECM- cells produce various cytokines and interleukins (*CXCL8*), ID+ cells are identified by strong expression of ID genes (*ID1*), and ECM+ cells by marked expression of ECM components (*COL1A1*). (**c**) The clusters are equivalent in JDF and PDF spheroids and display statistically significant differences in expression of their marker genes (** *p* < 0.01; *** *p* < 0.001, Mann-Whitney U test).

Analysis of scRNA-seq data revealed three different clusters of fibroblasts in both spheroid types (Figure 2). The clustering was similar in JDF and PDF spheroids, yet the cell groups were more notably separated in the PDF sample. The clusters differed in the expression of genes with well-described cellular functions: the first one was enriched in genes encoding inflammatory factors such as cytokines and chemokines (e.g., *CXCL8*) and depleted in genes responsible for production of the extracellular matrix (ECM). Thus, we denote the cluster as ECM^−^. The second cluster was enriched in *ID* genes responsible for differentiation/dedifferentiation processes that are included in the transforming growth factor β (TGF-β) signalling family cascade. We call this cluster ID^+^. The last cluster was enriched in ECM transcripts such as *COL1A1*, and thus we denote it ECM^+^.

**Figure 3:**
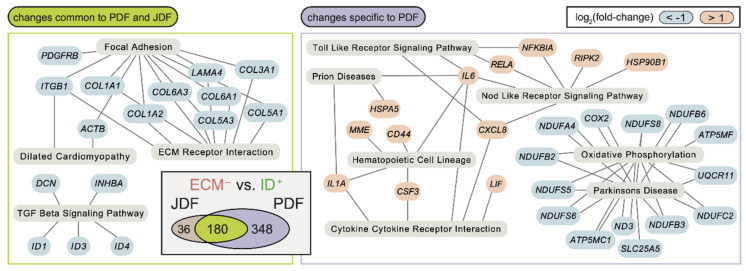
The differences between the ECM^−^ and ID^+^ fibroblast clusters are largely replicated in PDF and JDF spheroids, with distinct features present in PDF fibroblasts. Changes common to both samples (left) are enriched in genes downregulated in ECM^−^ clusters and participating in several KEGG pathways related to the extracellular matrix and TGF-β signalling. Changes specific to PDF spheroids (right) include hyperactivation of genes participating in cytokine signalling in the ECM^−^ cluster. No KEGG pathway enrichment specific to the JDF sample was observed. The Venn diagram (inset) displays a significant overlap (*p* < 10^−6^, Fisher’s exact test) of differentially expressed genes (false discovery rate FDR < 0.05, at least two-fold change in gene expression) in the comparison of the ECM^−^ and ID^+^ fibroblast clusters in JDF and PDF samples.

**Figure 4:**
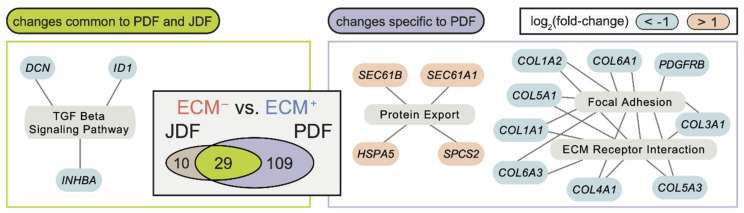
The differences between the ECM^−^ and ID^+^ fibroblast clusters are largely replicated in PDF and JDF spheroids with distinct features present in PDF fibroblasts. Changes common to both samples (left) are enriched in genes downregulated in ECM^−^ clusters and participating in several KEGG pathways related to extracellular matrix and TGF-β signalling. Changes specific to PDF spheroids (right) include hyperactivation of genes participating in cytokine signalling in the ECM^−^ cluster. No KEGG pathway enrichment specific to the JDF sample was observed. The Venn diagram (inset) displays a significant overlap (*p* < 10^−6^, Fisher’s exact test) of differentially expressed genes (false discovery rate FDR < 0.05, at least two-fold change in gene expression) in the comparison of the ECM^−^ and ID^+^ fibroblast clusters in JDF and PDF samples.

Gene expression differences between the clusters were to a large extent similar in JDF and PDF (Venn diagrams in Supplementary Figure S7). However, we observed some significant differences (Figure 3 and Figure 4 and heatmaps in Supplementary Figures S8 and S9). Comparison of the ECM^−^ to ID^+^ clusters (Figure 3 and Supplementary Table S2) revealed downregulation of genes associated with focal adhesion (KEGG hsa04510), ECM receptor interaction (hsa04512), and TGF-β signalling pathways (hsa04350) in both JDF and PDF. However, we observed a strong upregulation of genes associated with cytokine-cytokine receptor interaction (hsa04060), Nod-like receptor (hsa04621), and Toll-like receptor (hsa04620) signalling pathways in PDF only with prominent upregulation of the *CXCL8*, *IL6*, *IL1A*, and *LIF* genes (Figure 3). Furthermore, the downregulation of the genes in the oxidative phosphorylation (hsa00190) pathway was detected.

Similarly, we saw common differences in the comparison of ECM^−^ to ECM^+^ clusters (Figure 4): the TGF-β signalling pathway was significantly enriched for downregulated genes (*ID1*, *INHBA*, and *DCN*) in both JDF and PDF. In PDF, we observed a further enrichment of downregulated genes in the ECM receptor signalling pathway and focal adhesion, including several collagen types (e.g., *COL1A1*, *COL4A1*). We observed only marginal differences in the activity of the signalling pathways in the comparison of ID^+^ and ECM^+^ clusters.

**Figure 5:**
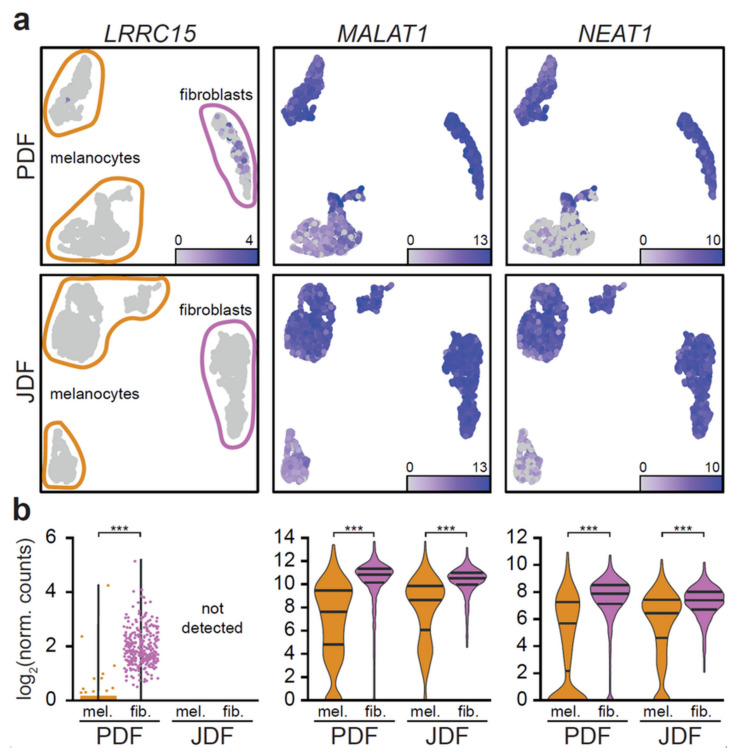
Expression of *MALAT1* and *NEAT1* distinguishes two melanoma cell clusters in each spheroid type. In UMAP projection of the data (**a**), LRRC15 (left) is expressed only in the ECM+ cluster of PDF (top) fibroblasts. *MALAT1* (centre), and *NEAT1* (right) display expression predominantly in the upper cluster of melanoma cells both in PDF (top) and JDF (bottom). (**b**) The expression intensities of the genes are different in fibroblasts and melanoma cells, with markedly bimodal distribution in melanoma cells (*** *p* < 0.001, Mann-Whitney U test).

These results indicated that the differences between fibroblasts caused by photoageing in PDF concentrate in the ECM^−^ clusters. To validate the hypothesis, we aggregated the scRNA-seq data from PDF and JDF using library size standardisation and compared ECM^−^ (ID^+^, ECM^+^ respectively) clusters between PDF and JDF spheroids. In all comparisons, we detected tens of differentially expressed genes (Supplementary Table S3 and Supplementary Figure S7 for their expression profile in aggregated data). Both ECM^+^ and ECM^−^ clusters displayed common upregulation of several genes in PDF (*TNFAIP6*, *TFPI2*, *ACKR3*, *DCN*, and *CTHRC1*). Specific changes in ECM^−^ clusters included upregulation of the genes linked to the TNF signalling pathway (hsa04668; *IL6*, *CCL20*, *CCL5*, *LIF*, *PTGS2*, and *MMP9*). These genes are often overexpressed in senescent cells. Changes between the ECM^+^ clusters involved upregulation of periostin *POSTN*, fibromodulin *FMOD*, and the gene coding for fibroblast activation protein *FAP*. Surprisingly, we observed the activation of the *LRRC15* (leucine-rich repeat containing 15) gene in ECM^+^ PDF fibroblasts, while no expression of *LRRC15* was detected in ECM^+^ JDF fibroblasts (Figure 5).

*CTHRC1* (collagen triple helix repeat containing 1) was upregulated in all PDF clusters compared to JDF, see Supplementary Table S3 and Supplementary Figure S7. We also observed downregulation of specific genes in ECM^−^ PDF fibroblasts (*RHOB*), in ECM^+^ PDF fibroblasts (*PGF* and *GAL*) and in ID^+^ PDF fibroblasts (*DUSP2* and *IGFBP2*) compared to their JDF counterparts. In all PDF clusters, we detected downregulation of the *MGP* gene, see Supplementary Figure S7.

In summary, while we found scattered changes in gene expression between PDF and JDF spheroids in ECM^+^ and ID^+^ clusters with the upregulation of genes coding for inflammation-supporting factors in the fibroblasts prepared from adult UV-irradiated skin, ECM^−^ clusters also displayed distinct upregulation of the TNF signalling pathway.

### 2.4. Bioinformatic Analysis of Melanoma Cells Isolated from Spheres Containing both Types of Fibroblasts

Using bioinformatic analysis of scRNA-seq data, we divided G361 melanoma cells from heterogeneous spheres to two clusters. In this case, we did not observe principally different transcriptional profiles. Instead, these clusters differed in transcriptional activity with one cluster more active than the other. The more active cells exhibited higher expression of *NEAT1*, *MALAT1* (Figure 5), *SERPINE2*, and *IGFBP3* genes. They also displayed a higher expression of *CDH1* and *CDH2* genes (Supplementary Figures S4 and S5).

### 2.5. Expression of Pro-Inflammatory Cytokines in Spheroid Components

**Figure 6:**
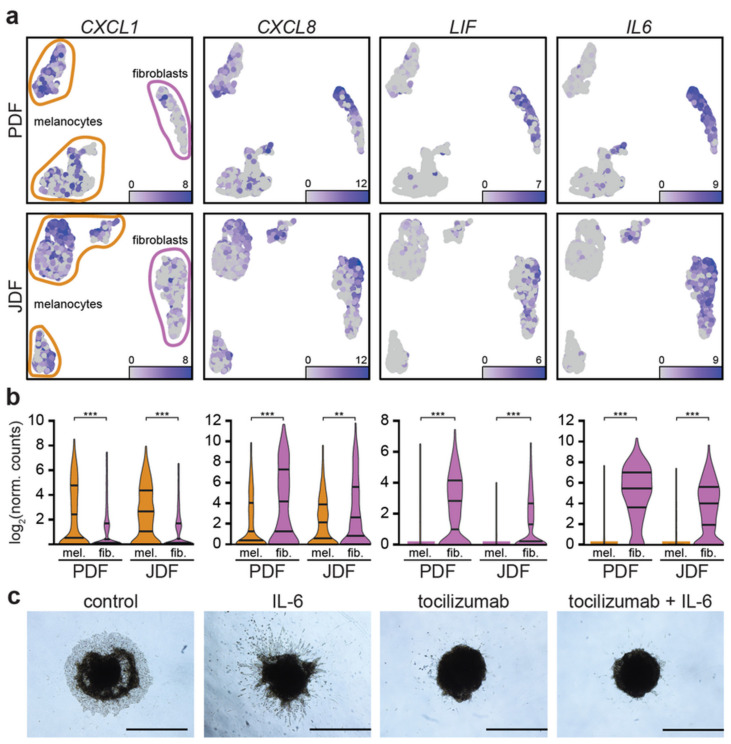
Expression of inflammation-related genes in different components of the PDF and JDF spheroids. In UMAP projection of the data (**a**), chemokine *CXCL1* (left) and interleukin *CXCL8* (centre left) are expressed both in melanoma cells and fibroblasts in PDF (top) and JDF (middle) spheroids. *LIF* (centre right) and interleukin *IL6* (right) are predominantly expressed in fibroblasts. The observed differences in gene expression are statistically significant (** *p* < 0.01; *** *p* < 0.001, Mann-Whitney U test). (**b**) The migratory behaviour of cells in a heterogenous spheroid is strongly stimulated by IL-6 and diminished upon tocilizumab treatment (**c**). Bar denotes 1000 μm.

As we observed the expression of pro-inflammatory cytokines in specific clusters of fibroblasts, we examined whether these factors were also expressed by melanoma cells. We observed that *CXCL1* was predominantly produced by the melanoma cells, while *IL6* and *LIF* were expressed mainly by the fibroblasts and *CXCL8* by both cell types (Figure 6a,b).

### 2.6. Spheroid Viability, Invasion to the Extracellular Matrix and Pharmacological Blockade of the IL-6 Receptor

The spheroids retained high viability of cells, as evidenced by trypan blue supravital staining performed after enzymatic digestion prior to RNA analysis (not shown). Moreover, both types of cells from the digested spheres were able to be read here and continue proliferation in 2D.

After embedding to the collagen matrix, heterogeneous spheroids presented clear cellular outgrowths as a sign of invasion. We observed a migration of individual fibroblasts followed by melanoma cells (Figure 6c, JDF presented here, similar PDF not shown). Their remarkably distinct morphology can easily distinguish these cell types. In controls with complete culture medium (DMEM 10% FBS), we observed the tightly packed invasive front of G361 melanoma cells. The invasive potential was remarkably enhanced by addition of exogenous IL-6 to the culture medium. The G361 invasion was more irregular and formed massive tentacular outgrowths in the full thickness of the collagen layer. We also observed individual G361 cell migration. The addition of tocilizumab to the culture medium significantly restricted the outgrowth of melanoma cells to the collagen gel. The rescue experiment where tocilizumab was applied simultaneously with IL-6 did not differ significantly from the earlier one. Invariably, the viability of spheroids even after ten days in the collagen matrix was confirmed by MTT test (Supplementary Figure S11). This suggested the sustained metabolic activity of spheroids in all experiments.

## 3. Discussion

As evidenced by our data, heterogeneous spheres containing fibroblasts and melanoma cells can be analysed by the scRNA-seq technique. This approach represents a very promising tool for deeper study of the cancer microenvironment organisation and function. scRNA-seq gives a unique chance to identify even a minor population within a seemingly homogeneous pool of stromal fibroblasts and reveal their regulatory function. Bioinformatic analysis allows shedding light on the combinations of well-established markers and identifying the previously unknown activity of a subset of genes in a defined population. It can facilitate study of the changes associated with the intercellular crosstalk in tumours in 3D.

In our experiments, both JDF and PDF fibroblasts from photodamaged skin exhibited marker of activated fibroblasts *FAP* (fibroblast activations protein) in heterogeneous spheroids with G361 melanoma cells. FAP is a cell-surface serine protease that acts on the extracellular matrix components and some other substrates. FAP is highly upregulated in a wide variety of cancers and it is often used as a marker for pro-tumorigenic stroma [34].

Fibroblasts were, according to their expression profile, assigned into three clusters. This observation shows that they do not represent the homogeneous population and that they can be distinguished according to their effect on the cancer cell. This also indicates a high complexity of the melanoma ecosystem.

Fibroblasts of the ECM^+^ cluster produced ECM, e.g., different types of collagen. This cluster represents an older paradigmatic view of fibroblasts as cells continuously contributing to the formation of scaffolds of ECM in both normal tissues and tumours. Notably, we observed activation of *LRRC15* in the ECM^+^ cluster of PDF fibroblasts. LRRC15 was described as a membrane-bound protein on stromal fibroblasts in many solid tumours (e.g., breast, head and neck, lung, pancreatic). The LRRC15 expression was induced by TGF-β on activated fibroblasts (αSMA^+^) and on mesenchymal stem cells [35]. In our case, LRRC15 was surprisingly detected in photodamaged, yet normal primary fibroblasts. This finding highlights the loose definition of CAFs and the contemporary lack of their specific markers [36,37,38,39].

When compared to other fibroblasts clusters, the ECM^−^ cluster cells displayed upregulated genes for production of inflammation-promoting factors, namely *IL6*, *CXCL8*, and *TGFβ*.

The signal was much stronger in the cells of the ECM^−^ cluster of the PDF population, which also overexpressed several other genes linked to the pro-inflammatory TNF signalling pathway (*IL6*, *CCL20*, *CCL5*, *LIF*).

These molecules have a potent regulatory effect and can promote tumorigenesis. Notably, there is an obvious overlap with molecules representing the senescence-associated secretory phenotype. Because of the prominent role of IL-6, we studied the effect of clinically approved antibody tocilizumab directed against the complex of IL-6 receptor with gp130 on the migration of cells from the spheres transferred to adhesive conditions. While both types of fibroblasts and melanoma cells can migrate out of the spheroids, the application of tocilizumab remarkably inhibited migration of melanoma cells (Figure 6c).

IL-8 seems to be another dominant player that we observed in the ECM^−^ cluster phenotype. IL-8 is also activated in senescent cells. In stress conditions, IL-8 confers chemotherapeutic resistance on cancer cells [40]. IL-8 signalling promotes angiogenic responses, proliferation, and survival of cancer cells, and potentiates their migration. Therefore, inhibiting the effects of IL-8 signalling may be a significant therapeutic intervention in targeting the tumour microenvironment. However, IL-8 targeting has reached rather modest clinical attention so far [41].

Chronic inflammation is a hallmark of senescence and represents a very important condition for initiation and progression of practically all types of malignant diseases, including melanoma. Beside immune cells, CAFs produce numerous factors that stimulate tumour growth [42]. Among factors that enhance cancer growth and spreading, both IL-6 and IL-8 play a fundamental role not only in cutaneous but also in uveal melanoma [15,43,44,45,46]. This observation can demonstrate how the model conditions can mimic the microenvironment. Further, employment of the presented system based on fibroblasts is important in the design and testing of potential new anticancer approaches.

It was observed in pancreatic cancer that IL-1 induces LIF expression and downstream JAK/STAT activation to generate inflammatory CAFs [47]. Moreover, TGF-β was suggested to antagonise this process by downregulating IL-1R1 expression and promoting differentiation into myofibroblasts. Therefore, it was suggested that distinct CAF subtypes are characterised by either myofibroblastic or inflammatory phenotypes.

TGF-β itself can have both tumour-suppressive (at an early stage) and tumour-promoting effects (at the late stage of carcinogenesis). TGF-β directly promotes the oncogenic potential of tumour cells and enhances tumour cell invasion and migration. This is achieved by driving epithelial-to-mesenchymal transition (EMT), a hallmark of cancer [48].

The ID^+^ cluster is also related to TGF-β in a broader sense. However, it must be seen independently from the previous one. Fibroblasts of this cluster had a prominent activity of *ID* (Inhibitor of Differentiation) genes. Members of the broad TGF-β family of ligands include the TGF-βs, activins, NODAL, BMPs, and GDFs. These molecules share pleiotropic effects on cell behaviour ranging from germ layer specification and patterning in embryonic development to tissue homeostasis and regeneration in adults [49].

The best characterised signalling pathway downstream of TGF- β is through SMAD2 and SMAD3 via their phosphorylation. However, there is also non-canonical signalling induced by TGF-β, which depends on SMAD1/5 phosphorylation. This somewhat surprising alternative was explained in recent years by a new paradigm for receptor activation where TGFBR1 phosphorylates and activates ACVR1 [50]. Primary early transcriptional targets of this axis are the *ID* genes typical of this cluster of our fibroblasts.

A similar extent of fibroblast heterogeneity was also observed by other authors, who confirmed the polarisation of normal dermal fibroblasts to those producing ECM and fibroblasts producing pro-inflammatory factors. Of note, the particular composition of the proposed clusters was age-dependent [51,52]. This was also demonstrated in our study, comparing JDF and PDF.

A heterogeneous population of CAFs was also observed in tumours, such as breast and prostate [53,54]. Production of collagen by CAFs and its elongation is even able to influence the prognosis of patients in many types of cancers [55].

Our model of heterogeneous spheroids demonstrated differences between fibroblasts prepared from a young person and an adult donor after chronic UV exposure, which is not surprising [2,52]. The population of PDF after years of UV chronic injury of donor skin seems to be more heterogeneous, which may be influenced by accumulated UV-dependent damage. Induction of a senescence-associated secretory phenotype may stimulate cancer growth to a greater extent than factors released from the young individual not damaged by irradiation. Such interpretation is supported by a classical study [56] that demonstrated the effect of the irradiation of fibroblasts on mammary gland cancer formation in animal experiments.

It is also noteworthy that we used JDF and PDF fibroblasts after significant in vitro quantity expansion (at passage 5). This is a generally accepted maximum passage number when working with early primary isolates, including fibroblasts. The clonal selection occurring in-vitro and its potential implications have long been discussed [56]. This phenomenon may have significant consequences for reliable disease modelling at a larger scale [57], because the natural cellular diversity seen in the tissue can be easily lost. This would lead to the underrepresentation of specific phenotypes in the models. Based on our observation, a certain diversity in fibroblast clustering remained preserved even in the earlier passages (no. 5). The mechanisms underlying the maintenance of their diversity remain unknown. Of note, we have earlier observed sustained biological activity in various cancer-associated fibroblast cultures even of significantly higher passages [31,58,59].

In melanoma cells, we identified two clusters in both types of spheres. We used the well-characterised cell line G361 for our melanoma 3D model. These cells have been immortalised and maintained in vitro for a substantially long time. The reason for clustering is, therefore, unlikely to represent any natural heterogeneity observed in tumours [60,61]. Melanoma is an aggressive disease, frequently adopting a more de-differentiated phenotype close to stemness and also similar to neural crest stem cells [62,63]. This low differentiation status is typical of many malignant melanoma cell lines. Emerging evidence indicates that both quiescent (out of cell cycle and in a lower metabolic state) and active (in cell cycle and not able to retain DNA labels) stem cell subpopulations may coexist, as described in several tissues [64,65].

As mentioned above, we observe a division of G361 cells into two clusters. The transcriptionally more active cluster of G361 in our spheroids exhibited an upregulation of *NEAT1*, *MALAT1* (also known as *NEAT2*), *SERPINE2*, and *IGFBP3* genes. Both *NEAT1* and *MALAT1* genes are affiliated with the lncRNA class. Recent studies have demonstrated implication of long noncoding RNAs (lncRNAs) in a variety of physiological and pathological processes. These genes are important for nuclear organisation because of their role in stabilisation of nuclear bodies and splicing of pre-mRNA. They stimulate migration of both types, i.e., cutaneous and uveal melanoma [66,67,68,69]. Their role in chemoresistance of cancer cells to therapeutics is discussed [70]. Therefore, their targeting by small inhibitors was recently proposed in the literature [71].

Mechanistically, it was suggested that the MALAT1-protein complex facilitates dephosphorylation of pSmad2/3 by providing the interaction niche for pSMAD2/3 and their specific phosphatase PPM1A, thus regulating TGF-β/SMAD signalling [72].

Similarly, the activity of *SERPINE2* stimulates the melanoma metastatic behaviour [73]. On the other hand, the role of the *IGFBP3* gene in cancer is not entirely understood, because both melanoma-supporting and inhibiting effect was shown concerning this factor [74,75]. The activity of genes encoding E- and N-cadherins (*CDH1*, *CDH2*) was detected in melanoma cells prepared from the spheroids. The presence of N-cadherin in these cells seems to biologically relevant because the switch of the expression of E-cadherin to N-cadherin enhances migration of cancer cells and stimulates cancer spreading [76]. This result harmonises well with the activity of noncoding transcripts *MALAT1* and *NEAT1*.

We demonstrated that spheroids constructed from melanoma cells and fibroblasts in combination with single-cell sequencing represent a suitable model for melanoma research that can be employed for the study of the interaction of fibroblasts with melanoma cells.

Our study confirmed that dermal fibroblasts are not homogeneous but instead comprise multiple discrete subpopulations with extensive variations confirmed by our scRNA-seq data. Since fibroblasts are the predominant cell type in dermal tissue, the regulation of their behaviour is likely to be important in tissue physiology and pathology. We believe that these variations documented by scRNA-seq are relevant to our understanding of skin biology and their involvement in dermatological diseases, including melanoma. We hypothesize that more in-depth knowledge of fibroblast heterogeneity represents an important benefit and can impact on the development of new approaches to therapy. As fibroblasts are the most ubiquitous cells found in the dermis and various other tissues, therapeutic targeting of this cell type, in general, seems to be a risky and unrealistic goal for future therapy. It is more likely that the targeting of a discrete population with distinct protumorigenic behaviour would be more plausible and could be associated with a clinical benefit.

In the nearest future, it must also be verified how these heterogeneous models could be subjected to various external stimuli, including pharmacological treatment with, e.g., BRAF and MEK inhibitors. We believe that our presented findings would allow us to interpret these models realistically and overcome the current limitations of our study.

## 4. Materials and Methods 

### 4.1. Cell Isolation and Culture

Skin specimens were obtained during the routine surgery at the Department of Dermatology (n = 10), Charles University, Prague. The samples of residual skin were collected under the Local Ethics Committee approval in accordance with the ethical standards of the Institutional and National Research Committee, and according to the 1964 Helsinki declaration and its later amendments or comparable ethical standards. Informed consent from individual participants and/or representatives was obtained.

The fibroblasts were isolated from skin explants according to a protocol published earlier by us and immunophenotyped [77]. Briefly, fibroblasts were expanded in Dulbecco’s Modified Eagle’s Medium (DMEM), supplemented with 10% foetal bovine serum (both from Biosera, Nuaille, France) with antibiotics (penicillin 100 IU/mL, streptomycin 100 μg/mL and gentamycin 100 μg/mL, all Sigma Aldrich, Prague, Czech Republic) (FBS, Biosera, Nuaille, France). For the scRNA-seq study, two isolates of fibroblasts were used. PDF (photoaged dermal fibroblasts) were isolated from the heavily photodamaged skin of a 55-year-old male. JDF (juvenile dermal fibroblasts) were isolated from the sun-protected skin of a 3-year-old boy. In both cases, the skin samples were clinically normal and devoid of any clinical signs of skin disease. For this experiment, we used early passages before P4-5. The cells were well and fast growing and were devoid of β-galactosidase reaction (at pH = 6, less than 1% subconfluent cells). The cell line of cutaneous malignant melanoma G-361 harbouring BRAF mutation (heterozygous for BRAF p.Val600Glu—c.1799T > A), was used for our study [78,79]. Cell authentication was performed before the experiment according to ISO/IEC 17025. The cells were maintained in Mc Coy’s Medium (Biosera, Nuaille, France) supplemented with antibiotics and 10% FBS. For further experiments with fibroblasts, G361 was grown in DMEM as above. All cells were routinely screened for mycoplasma infection.

### 4.2. Preparation of Heterogeneous Spheres 

The spheroids were prepared by the hanging drop method [80]. Briefly, fibroblasts were mixed in suspension with G-361 cells in a 1:1 ratio in complete culture medium. Individual drops (25 μL each) containing 50,000 cells were placed on the inner non-adhesive side of a 100 mm Petri dish lid (Corning Life Sciences, Tewksbury, MA, USA). Then, the Petri dish bottom part was filled with 15 mL of Dulbecco’s Phosphate-Buffered Saline (PBS, Biosera, Nuaille, France). The lid with drops was gently reverted and placed on the top. The hanging drops (approximately 50 per lid) were kept undisturbed in the incubator (37 °C, 5% CO_2_) for 60 h. Using a Pasteur pipette, the spheres were collected and moved to a new, sterile, bacteriological, non-adherent Petri dish with DMEM with 10% FBS and kept floating for an additional 48 h in the incubator.

### 4.3. Immunohistochemistry 

The spheroids were collected and gently rinsed in PBS, and the fixation and embedding followed the AMeX technology described elsewhere [81]. The human melanoma samples were fixed in 10% neutral buffered formalin and routinely processed in paraffin blocks (FFPE). The FFPE samples were sectioned (5 μm) and attached on positively charged X-tra adhesive slides (Leica, Bretton, UK). Heat-induced epitope retrieval was performed in DAKO Envision Flex buffer with endogenous peroxidase quenching using 1% hydrogen peroxide. Primary antibody incubation was performed overnight at 4 °C. The antibodies and immunohistochemical detection kit are listed in Supplementary Table S1. The photodocumentation was performed with a Leica DM2000 microscope using LASx software.

### 4.4. Single-Cell RNA Sequencing

For scRNA-seq analysis, 50 spheroids of each type (G-361 + PDF and G-361 + JDF, respectively) were used. The spheroids were collected and washed twice with PBS followed once by 0.02% EDTA solution. To prepare a single-cell suspension, we used a 1:1 mixture of 0.25% trypsin and 0.02% EDTA solution (all Sigma Aldrich, Prague, Czech Republic) at room temperature for approximately 10 min with gentle agitation. The viability of cells was assessed by trypan blue and counted in an automated TC20 cell counter (BioRad, Prague, Czech Rep.). Both samples had cell viability above 80%. Single-cell RNA-seq libraries were prepared using the Chromium controller instrument and Chromium next gem single-cell 3′ reagent kit (version 3.1) according to the manufacturer’s protocol (both 10× Genomics, Pleasanton, CA, USA) targeting at 4000 cells per sample. The quality and quantity of the resulting cDNA and libraries was determined using Agilent 2100 Bioanalyzer (Agilent, Santa Clara, CA, USA). The libraries were sequenced in two runs of NextSeq 500 sequencer using NextSeq 500/550 high output kit v2.5 (75 cycles) (both Illumina, San Diego, CA, USA) according to the manufacturer’s protocol.

### 4.5. Bioinformatic Analysis of scRNA-seq Data

Raw sequencing data were processed by CellRanger software v3.1.0 (10× Genomics, Pleasanton, CA, USA). The resulting raw feature barcode matrices were analysed in the R/Bioconductor statistical environment [82,83]. Empty droplets containing only ambient RNA were removed using DropletUtils [84]. Subsequently, dead or damaged cells were filtered out, resulting in 3908 and 4507 cells in the PDF and JDF sample, respectively. Features expressed in less than 5% of cells were removed from the analysis, giving a total number of 11,818 and 10,965 detected features in the PDF and JDF sample, respectively. The data were normalised, log_2_-transformed, and highly variable genes (HVGs) were detected. HVGs were used for the principal component analysis, which was used for the dimensionality reduction using Uniform manifold approximation and projection (UMAP) [85]. Single-cell consensus clustering (SC3) [86] was performed. The selected number of clusters was in accordance with the UMAP embedding of the cells.

Cluster marker genes were detected by the Mann–Whitney U test. The marker genes were required to be statistically significant (false discovery rate (FDR) < 0.05) and to have at least two-fold change in expression intensity between the clusters. The marker genes were subsequently used in the gene set enrichment analysis [87] of KEGG pathways [88]. The normalised enrichment score (NES) was used to account for differences in the gene set sizes. The thresholds for significantly enriched pathways were FDR < 0.005 and |NES| > 2.25. For analysis of the aggregated fibroblast dataset, the data of fibroblast subgroups within each sample were corrected for sequencing depth, integrated by mutual-nearest-neighbour algorithm, and clustered using SC3.

For the full description of bioinformatic analysis, see Appendix A [82,83,84,85,86,87,88,89,90,91,92,93,94,95,96,97].

### 4.6. Migration of Melanoma Cells from the Spheroids and Viability Assessment

To test the extracellular matrix invasion, we first coated the plates with a thin layer of 1% low melting point agarose (Promega, Madison, WI, USA). Next, we transferred the spheroids (6 per 10 cm^2^ well) onto agarose and overlaid them with freshly neutralised 2 mg/mL pre-cooled solution of collagen-1 (Gibco, Thermo Fisher, Waltham, MA, USA) following the manufacturer’s protocol. After collagen polymerisation (approximately 5 min at 37 °C), the complete culture medium was gently added dropwise. The culture medium was changed every 48 h afterwards. To stimulate ECM invasion, we supplemented the culture medium with human recombinant IL-6 (Sigma Aldrich, Prague, Czech Republic) at a final concentration of 10 ng/mL [98]. To block the IL-6 signalling cascade, we used monoclonal antibody tocilizumab (Roche, Grenzah-Wyhlen, Germany) at a final concentration of 10 ug/mL [98]. For the rescue experiment, we used the combination of IL-6 and tocilizumab simultaneously. DMEM + 10% FBS-treated spheroids were used as a control. The spheroids were maintained at 37 °C, 5% CO_2_ in a humidified incubator. Photos of migrating cells were taken every 24 h using a phase-contrast microscope. At day 10, we performed the final spheroid viability assessment using the MTT test. The medium was supplemented with MTT (3-(4,5-dimethylthiazol-2-yl)-2,5-diphenyl tetrazolium bromide (Sigma Aldrich, Prague, Czech Republic) at a final concentration of 1 mg/mL for 60 min, and insoluble formazan production in cells was assessed microscopically.

## 5. Conclusions

Heterogeneous spheroids combining cancer cells and other cell populations forming the cancer ecosystem (as exemplified here on fibroblasts) represent a highly relevant and still affordable model for cancer research. The combination with scRNA-seq allows deciphering the complex interactions in the heterogeneous spheroids. scRNA-seq is an excellent tool to understand the intercellular interactions in cancer under defined conditions significantly closer to the real situation than conventional cell cultures. Extrinsic and intrinsic stimuli shape the tissue microenvironment and can contribute to the establishment of a cancer-promoting niche. Fibroblasts are critical players in establishing the pro-inflammatory environment surrounding tumour cells. We have demonstrated that fibroblasts are functionally heterogeneous in spheroids. This aspect of their heterogeneity should be carefully investigated in the future. Based on our findings, targeting distinct subpopulations within the cancer microenvironment seems to be a plausible approach to the development of novel anticancer therapeutics.

## Supplementary Materials

The following are available online at https://www.mdpi.com/2072-6694/12/11/3324/s1, **Supplementary Figure S1****:** Single-cell RNA-seq profiling separates melanoma cell from fibroblasts. (a) Markers of fibroblasts (FAP) and melanoma cells (melan-A) distinguish cellular types in the tumour microenvironment. Bar denotes 100 μm. M denotes malignant melanoma cells, S denotes surrounding stroma, yellow arrowheads show FAP-positive stromal fibroblasts (in red). (b) Identical markers used to identify clusters of fibroblasts (*FAP*) and melanoma cells (*MLANA*) in UMAP visualisation of the scRNA-seq data. (c) There is virtually no expression of *FAP* in melanoma cells and *MLANA* in fibroblasts (*** p < 0.001, Mann-Whitney U test). **Supplementary Figure S2:** Clusters of melanoma cells and fibroblasts identified in scRNA-seq data express typical lineage markers. In the heatmap, expression intensity of the top fifteen specific genes for each cluster are plotted in the PDF (top) and JDF spheroid (bottom). **Supplementary Figure S3:** Read coverage for the *CDKN2A* gene. The melanoma cell line G361 is characterised by truncating deletion of the *CDKN2A* gene at its 3′ end, which leads to translational failure. Position on chromosome 9 is shown in (a). Separate coverage tracks are shown for melanoma and fibroblast cells within PDF and JDF samples (b). The gene model for the *CDKN2A* gene is given in (c). **Supplementary Figure S4:** Expression intensity of selected markers in the PDF spheroid. Melanoma markers (tyrosinase *TYR* and *MITF*) are expressed in melanoma cells (two left clusters). Cadherins *CDH1* and *CDH2* show complementary activity in the active melanoma cell cluster in the UMAP projection. Vimentin (*VIM*) is expressed in both cell populations. Fibroblast markers collagen *COL1A2*, lysyl oxidase (*LOX*), podoplanin (*PDPN*), and *S100A4* are expressed predominantly in the right cell cluster, which consists of fibroblast cells. **Supplementary Figure S5****:** Expression intensity of selected markers in the JDF spheroid. Melanoma markers (tyrosinase *TYR* and *MITF*) are expressed in melanoma cells (two left clusters). Cadherins *CDH1* and *CDH2* show complementary activity in the active melanoma cell cluster in the UMAP projection. Vimentin (*VIM*) is expressed in both cell populations. Fibroblast markers collagen *COL1A2*, lysyl oxidase (*LOX*), podoplanin (*PDPN*), and *S100A4* are expressed predominantly in the right cell cluster, which consists of fibroblast cells. The small upper cluster displays markers of both fibroblasts and melanoma cells and most probably consists of cell duplets. It was disregarded in all present analyses. **Supplementary Figure S6****:** Immunohistochemical analysis of melanoma primary tumours and metastases. Tyrosinase was highly positive in primary tumours and metastases, the stroma was negative. E-cadherin and N-cadherin staining was of weak-to-moderate intensity in melanoma cells as well. The vimentin signal was strong both in tumour cells and in stromal fibroblasts. Podoplanin was negative in either melanoma cells or stromal fibroblasts. **Supplementary Figure S7****:** Venn diagrams displaying overlap of the upregulated (left) and downregulated (right) genes in ECM^-^ (or ID^+^ and ECM^+^) fibroblast clusters across JDF and PDF spheroids. Only genes with statistically significant (false discovery rate FDR < 0.05) and at least two-fold change in gene expression are shown. See Additional Table S3 for details. **Supplementary Figure S8**: Heatmap of expression intensities of the markers of three fibroblast clusters identified in the JDF spheroid. ECM^-^, ID^+^, and ECM^+^ clusters are clearly identified by expression of chemokines, ID genes, and components of extracellular matrix, respectively. **Supplementary Figure S9**: Heatmap of expression intensities of the markers of three fibroblast clusters identified in the PDF spheroid. ECM^-^, ID^+^, and ECM^+^ clusters are clearly identified by expression of chemokines, ID genes, and components of extracellular matrix, respectively. **Supplementary Figure S10:** Differences in expression between ECM^-^ and ID^+^ (left), ECM^-^ and ECM^+^ (centre), and ID^+^ and ECM^+^ (right) are largely similar in PDF (top) and JDF spheroids (bottom). The Venn diagrams display overlap of differentially expressed genes (false discovery rate FDR < 0.05, at least two-fold change in gene expression) in each comparison. **Supplementary Figure S11:** The migratory behaviour of cells in a heterogenous spheroid in 3D gels is strongly stimulated by IL-6 and diminished upon tocilizumab treatment. However, these differences were remarkable; all spheroids invariably retained good viability as visualised by formation of the purple-blue formazan product in MTT test after 10 days of the experiment. Bar denotes 200 μm. **Supplementary Table S1:** Antibodies used in the study and tumour sample-related details. **Supplementary Table S2**: Differentially expressed genes (DEG) between identified fibroblast clusters within JDF and PDF spheroids. Only DEG with statistically significant (false discovery rate FDR < 0.05) and at least two-fold change in gene expression are given. **Supplementary Table S3:** Differentially expressed genes (DEG) between identified fibroblast clusters across JDF and PDF spheroids. Only DEG with statistically significant (false discovery rate FDR < 0.05) and at least two-fold change in gene expression are given. The data were standardised by library size normalisation. **The dataset supporting the conclusions of this article is available in the ArrayExpress repository, [E-MTAB-9216, https://www.ebi.ac.uk/arrayexpress/experiments/ E-MTAB-9216]**.

## Appendix A. Supplementary Details of Bioinformatic Analysis of scRNA-seq Data

### Appendix A.1. Preprocessing of Raw Data

Raw sequencing data were processed by CellRanger software v3.1.0 (10× Genomics, Pleasanton, CA, USA). The resulting raw feature barcode matrices were analysed in the R/Bioconductor statistical environment. Empty droplets containing only ambient RNA were detected using the DropletUtils package. Subsequently, dead or damaged cells were filtered out: filtering of PDF cells was based on median absolute deviation (MAD) of the following cell metrics: total read counts, number of detected features, and percentage of mitochondrial and ribosomal features. A value was considered an outlier if it was more than three MADs from the median (downwards for total read counts and number of detected features, and upwards for percentage of mitochondrial and ribosomal features). Filtering based on MAD resulted in 3908 cells in the PDF sample. MAD filtering with the same threshold was not suitable for the JDF sample. After investigation of cell metrics (data not shown), several per cell filtering thresholds were chosen: minimum and maximum unique molecule counts of 1000 and 50,000, respectively, minimum of 1000 features detected, maximum of 20 % of mitochondrial features, and minimum of 10% of ribosomal features. After the application of these rules, 4507 JDF cells remained. Features expressed in less than 5% of cells were filtered out, giving a total number of 11,818 and 1965 detected features for PDF and JDF samples, respectively.

### Appendix A.2. Data Normalisation

Cell-specific biases were normalised using the deconvolution strategy for scaling normalisation implemented in the computeSumFactors function from the scran package. Normalised expression values were calculated using the logNormCounts method from the scater package. The expression values can be interpreted as log2-transformed normalised counts and can be used for clustering or dimensionality reduction.

### Appendix A.3. Selection of Highly Variable Genes (HVGs)

To identify HVGs, the squared coefficient of variation (CV2) for each gene was computed within scran and a minimal CV2 value of 1.5 was used as the threshold. This resulted in 2054 and 712 HVGs found in the PDF and JDF samples, respectively.

### Appendix A.4. Dimensionality Reduction

Prior to downstream analysis, principal component analysis (PCA) was performed using HVGs. Based on the elbow plots, the first six and eight principal components (PC) were chosen for PDF and JDF samples, respectively, as the ones explaining most of the variance in the data. The selected PCs were used for the dimensionality reduction using the uniform manifold approximation and projection (UMAP) method implemented in scatter.

### Appendix A.5. Cell Clustering

Single-cell consensus clustering (SC3) was performed with a different number of cell target clusters. Based on the subsequent analysis of cluster marker genes, two clusters were chosen as appropriate to distinguish between melanocytes and fibroblasts. The selected number of clusters was in accordance with the UMAP embedding of the cells.

### Appendix A.6. Cluster Marker Genes and Gene Set Enrichment Analysis (GSEA)

Cluster marker genes between melanocyte and fibroblast clusters were detected by the Mann–Whitney U test implemented in the FindAllMarkers function from Seurat v3 package. The marker genes were required to be statistically significant (false discovery rate (FDR) < 0.05) and to have at least two-fold change in expression. The marker genes were subsequently used in GSEA (53) on KEGG pathways using the clusterProfiler package. The normalised enrichment score (NES) was used to account for differences in the gene set sizes. The thresholds for significantly enriched pathways were FDR < 0.005 and |NES| > 2.25.

### Appendix A.7. Division of Data to Melanocyte and Fibroblast Subgroups

According to the UMAP embedding, the cells were divided to melanocyte and fibroblast subgroups. Data normalisation, HVG selection, dimensionality reduction, cell clustering, cluster marker genes, and GSEA were repeated in the independent subgroups. In the case of SC3 clustering, two and three clusters were selected as appropriate for melanocyte and fibroblast cells, respectively.

### Appendix A.8. Aggregation of Fibroblast Subgroups of the PDF and JDF Samples

The data of fibroblast subgroups within each sample were corrected for sequencing depth by the multiBatchNorm function from the batchelor package. The corrected data were integrated by the mutual-nearest-neighbour algorithm implemented in the fastMNN function from the same package, and SC3 was used for clustering. In this case, three target clusters yielded inappropriate results, with one of the clusters being split in parts (data not shown). Thus, four target clusters were chosen, where one of the clusters contained a low number of cells and was disregarded. The obtained cell-cluster assignments were used together with the data corrected for sequencing depth for the computation of cluster marker genes and GSEA.
