# Supplementary figures and images for: Single-Cell RNA Sequencing Unravels Heterogeneity of the Stromal Niche in Cutaneous Melanoma Heterogeneous Spheroids
Source: Cancers (Basel). 2020 Nov 10;12(11):3324. doi: 10.3390/cancers12113324 (PMC7697260; doi:10.3390/cancers12113324)

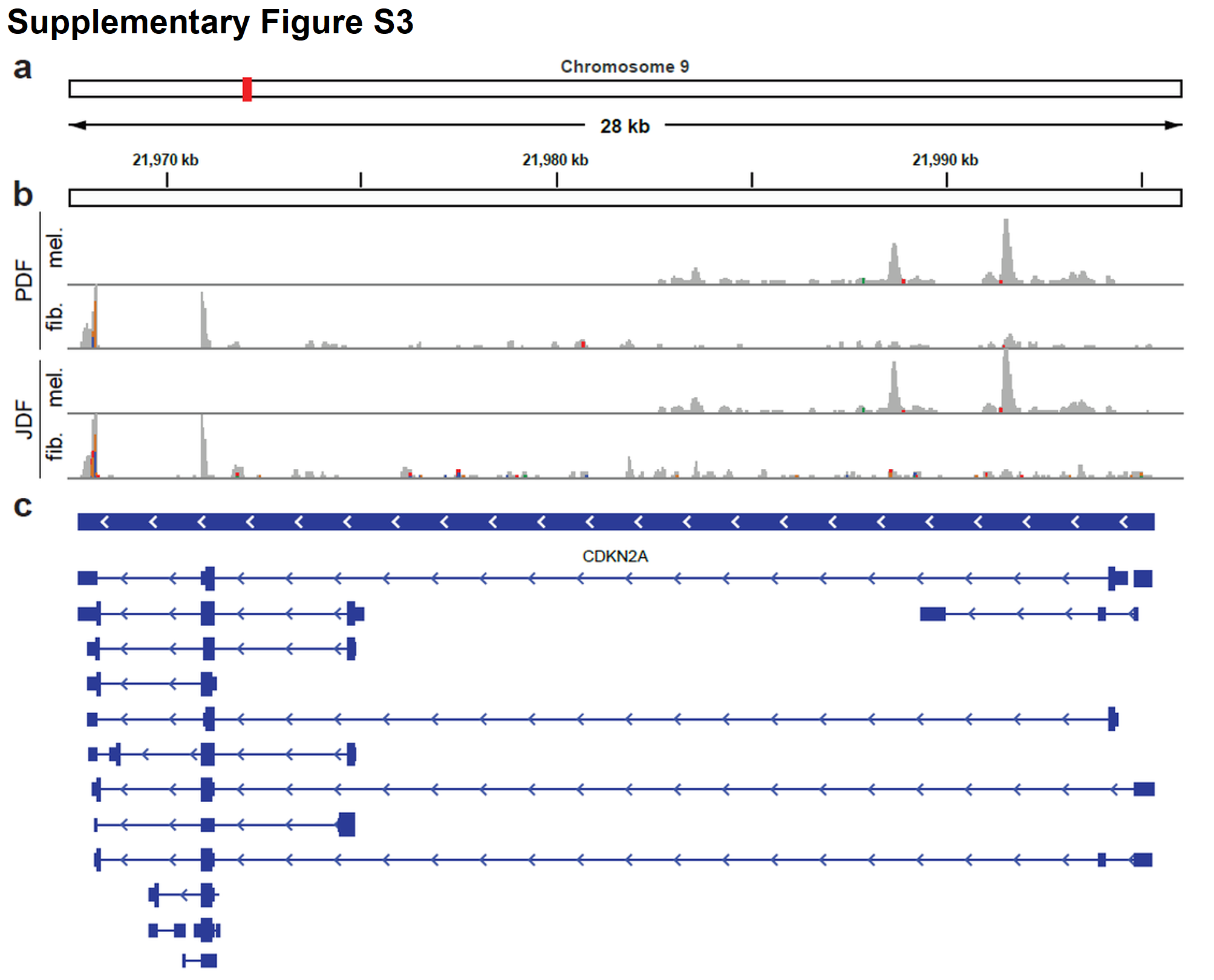

Supplement: Supplementary file 1 [file cancers-12-03324-s001.zip › Supplementary Figure S3.PNG]

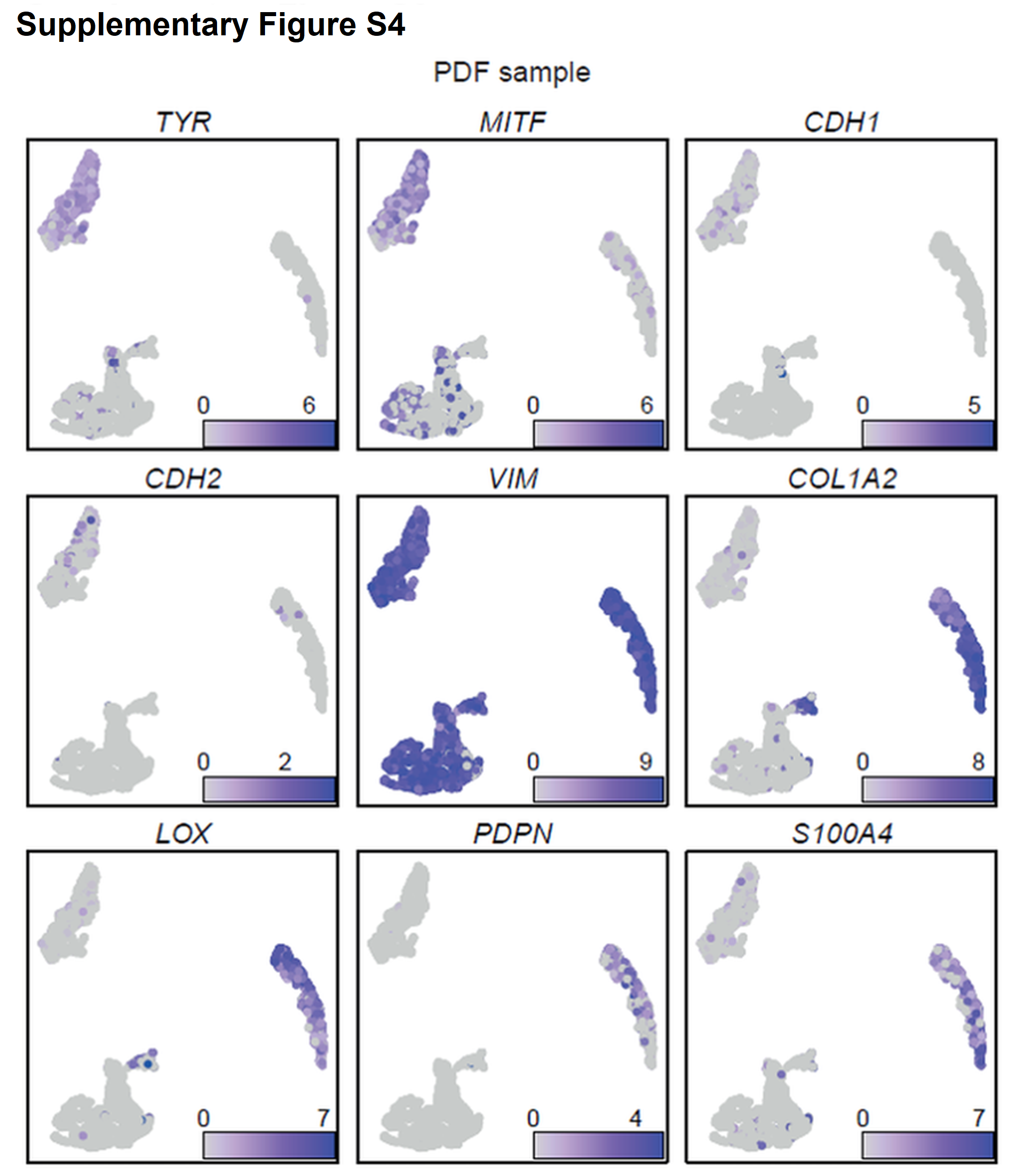

Supplement: Supplementary file 1 [file cancers-12-03324-s001.zip › Supplementary Figure S4.PNG]

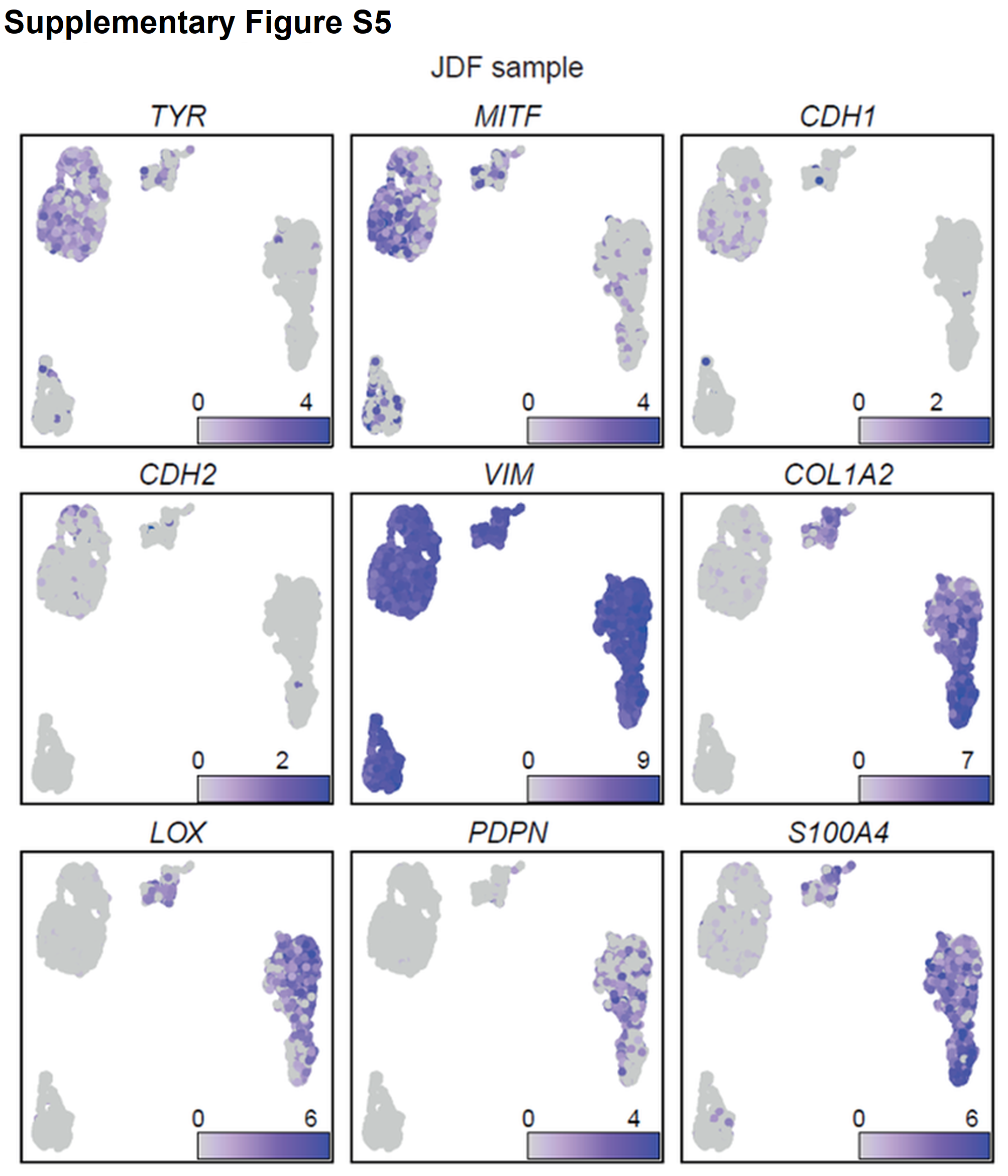

Supplement: Supplementary file 1 [file cancers-12-03324-s001.zip › Supplementary Figure S5.PNG]

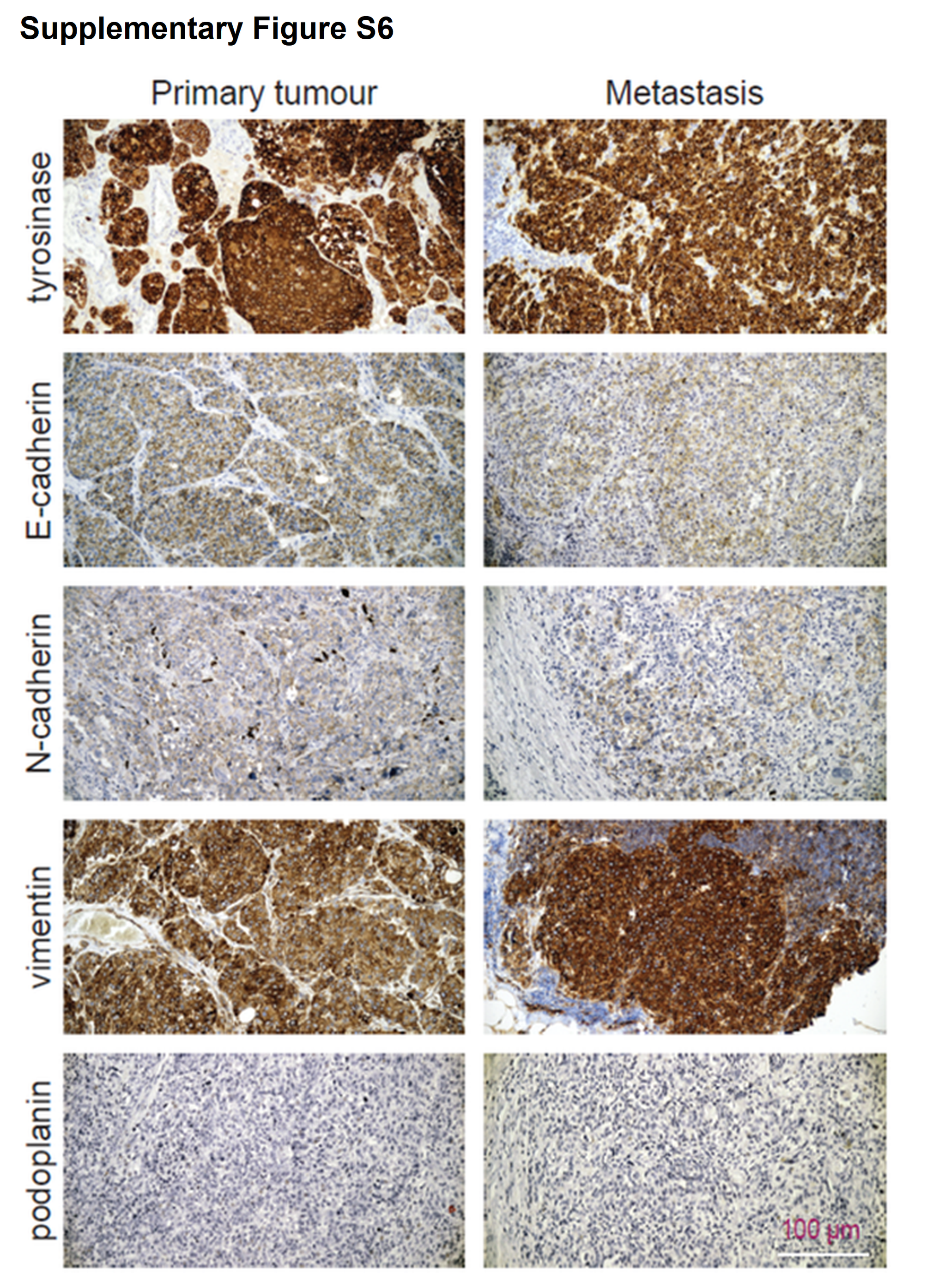

Supplement: Supplementary file 1 [file cancers-12-03324-s001.zip › Supplementary Figure S6.PNG]

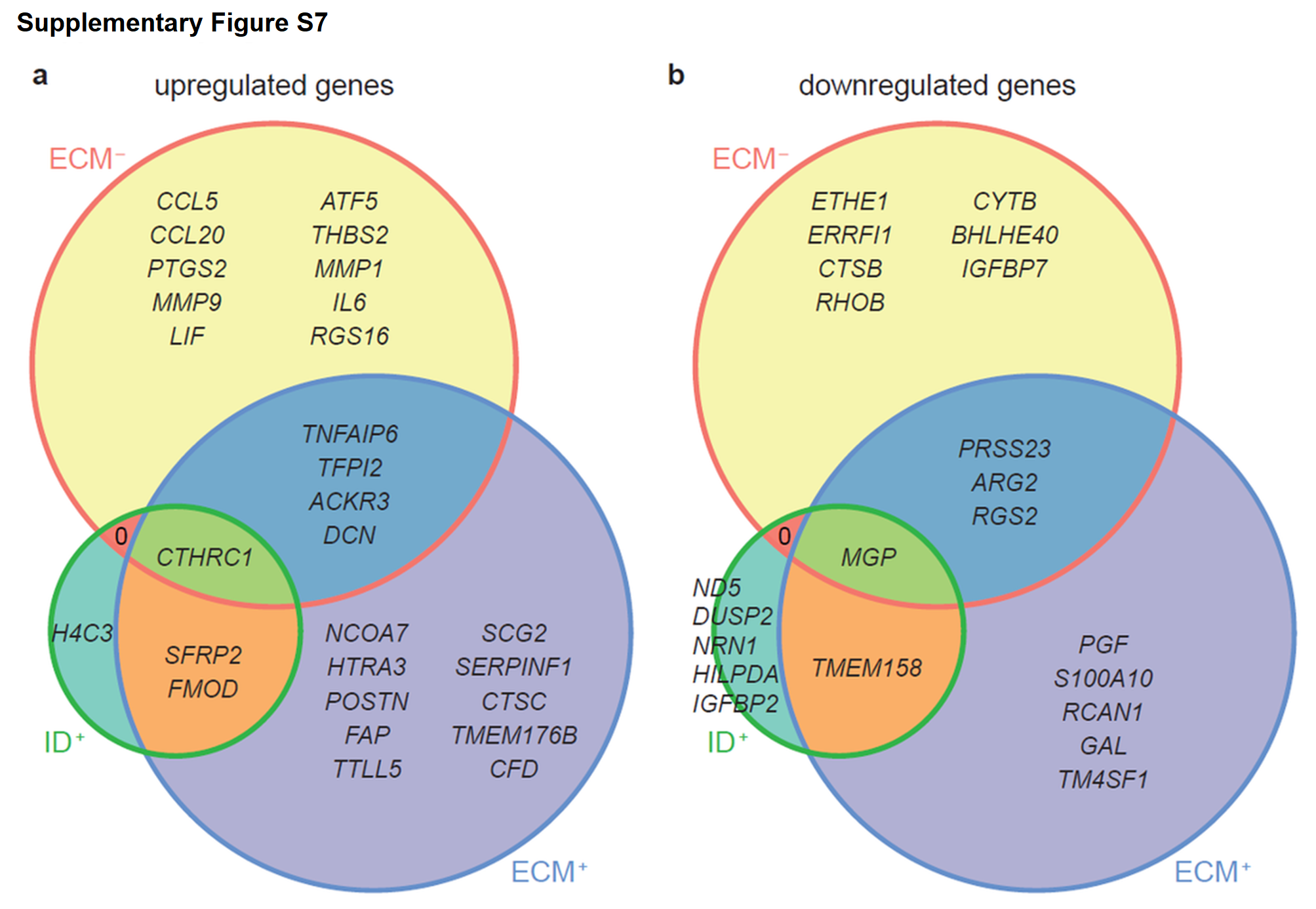

Supplement: Supplementary file 1 [file cancers-12-03324-s001.zip › Supplementary Figure S7.PNG]

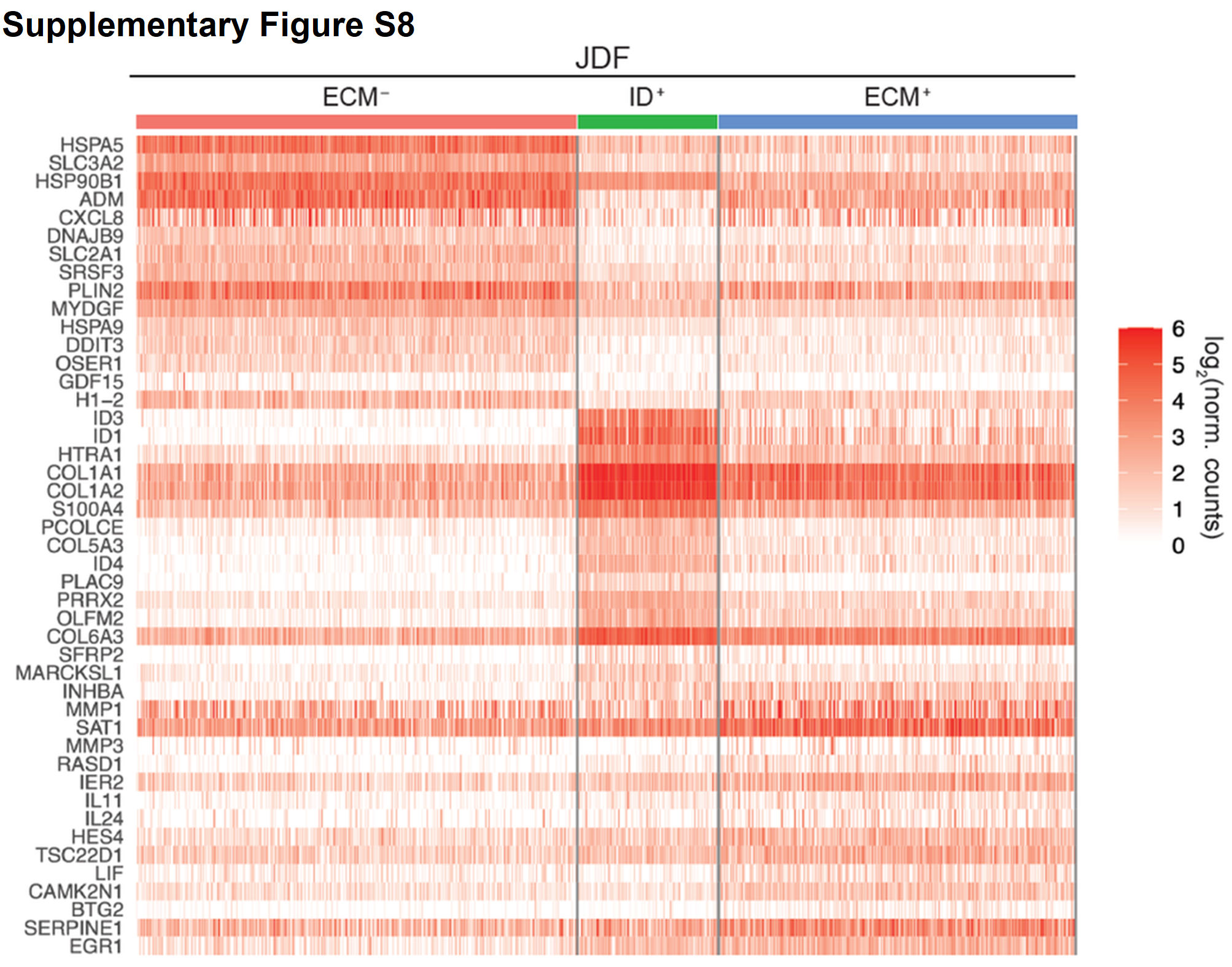

Supplement: Supplementary file 1 [file cancers-12-03324-s001.zip › Supplementary Figure S8.PNG]

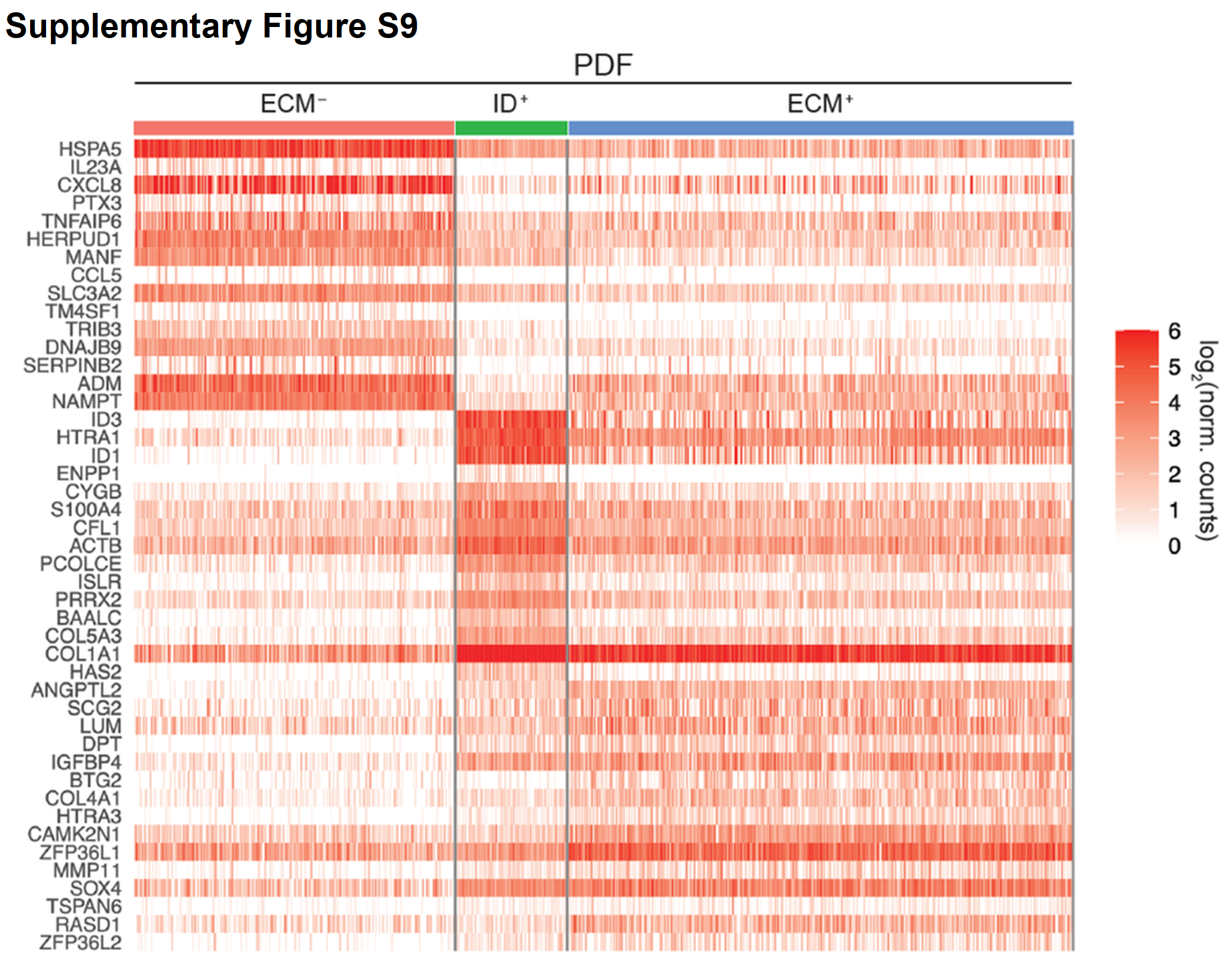

Supplement: Supplementary file 1 [file cancers-12-03324-s001.zip › Supplementary Figure S9.PNG]

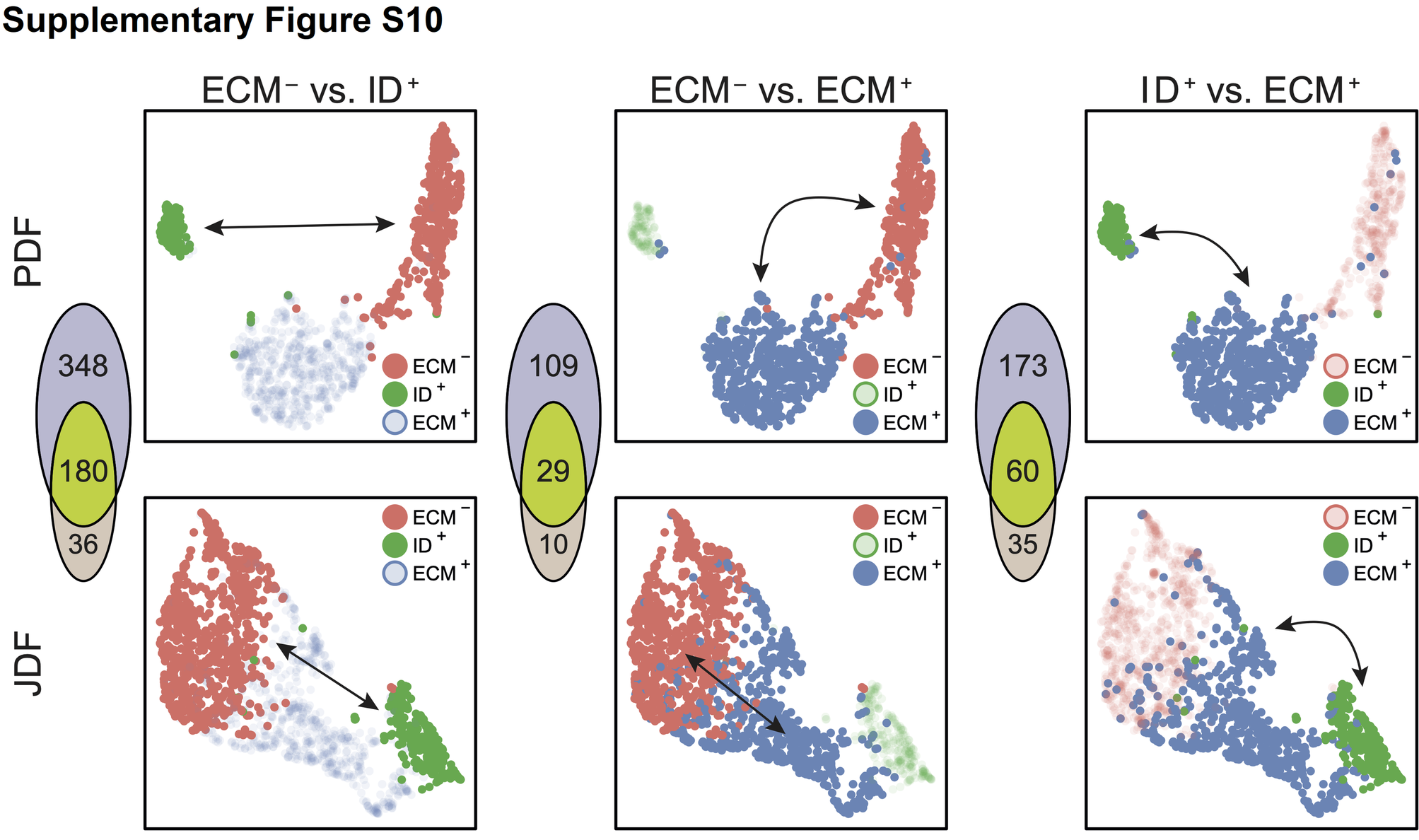

Supplement: Supplementary file 1 [file cancers-12-03324-s001.zip › Supplementary Figure S10.png]

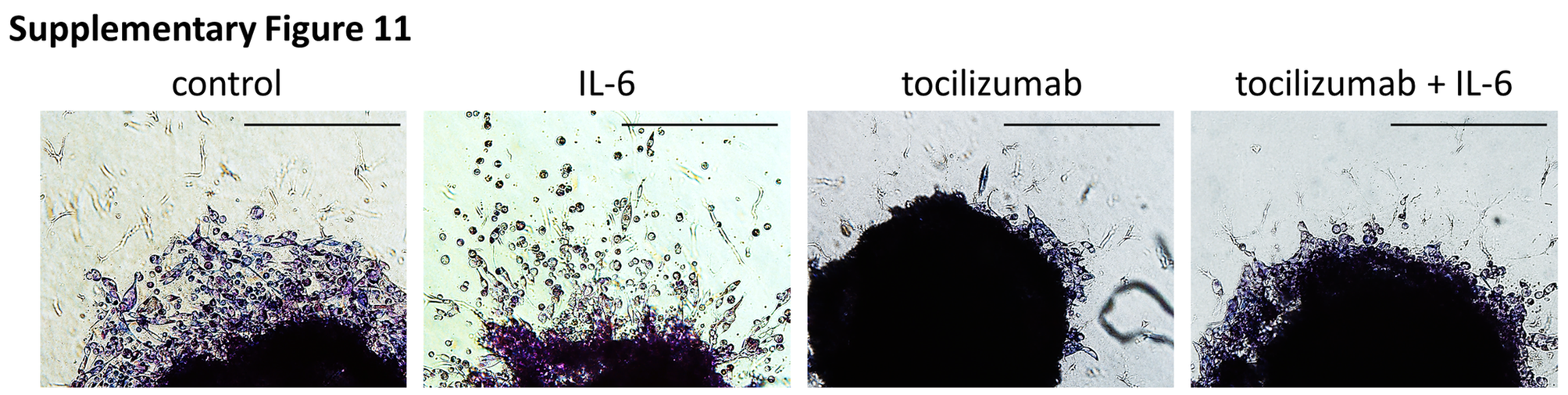

Supplement: Supplementary file 1 [file cancers-12-03324-s001.zip › Supplementary Figure S11.png]

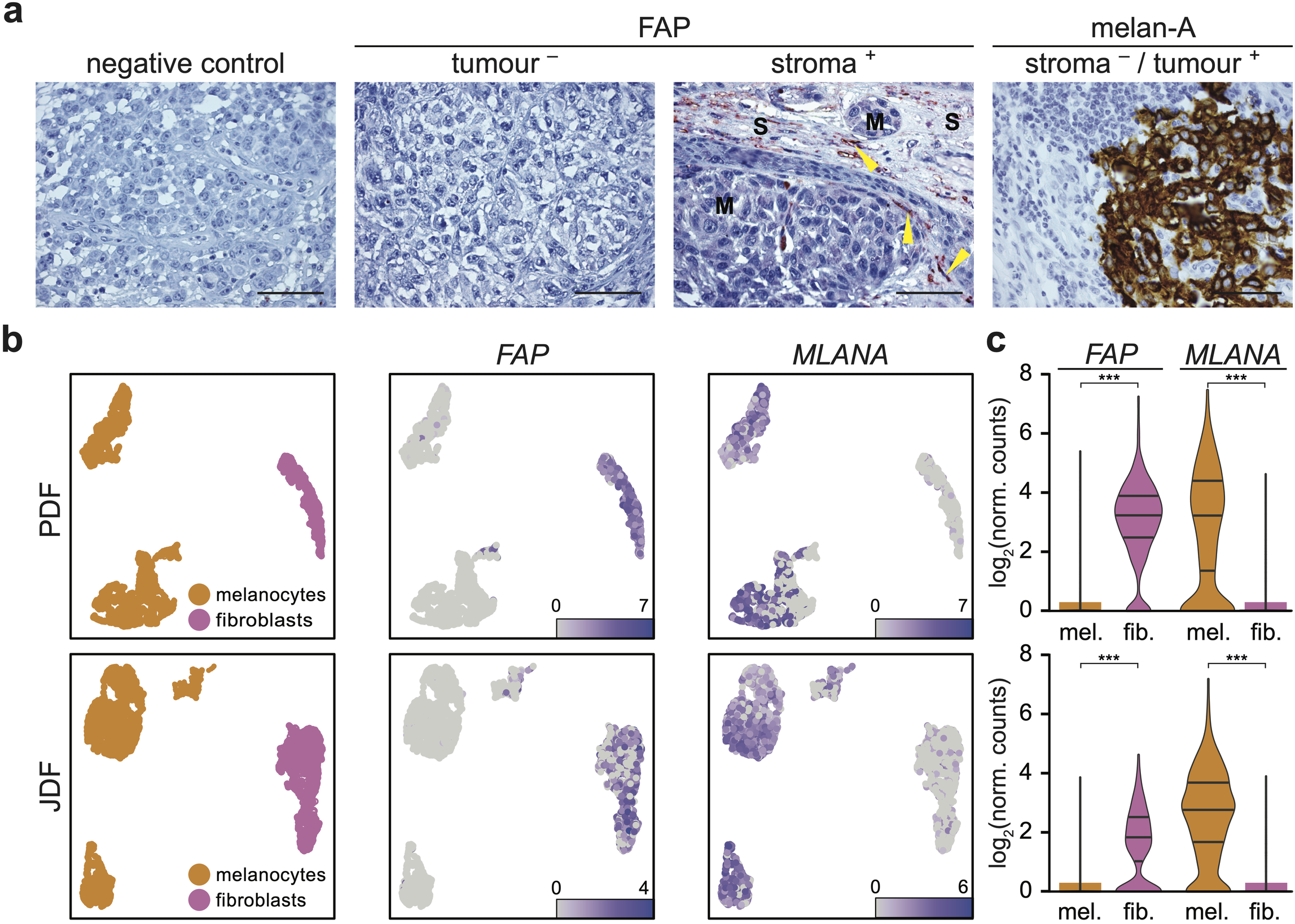

Supplement: Supplementary file 1 [file cancers-12-03324-s001.zip › Supplementary Figure S1.tiff]

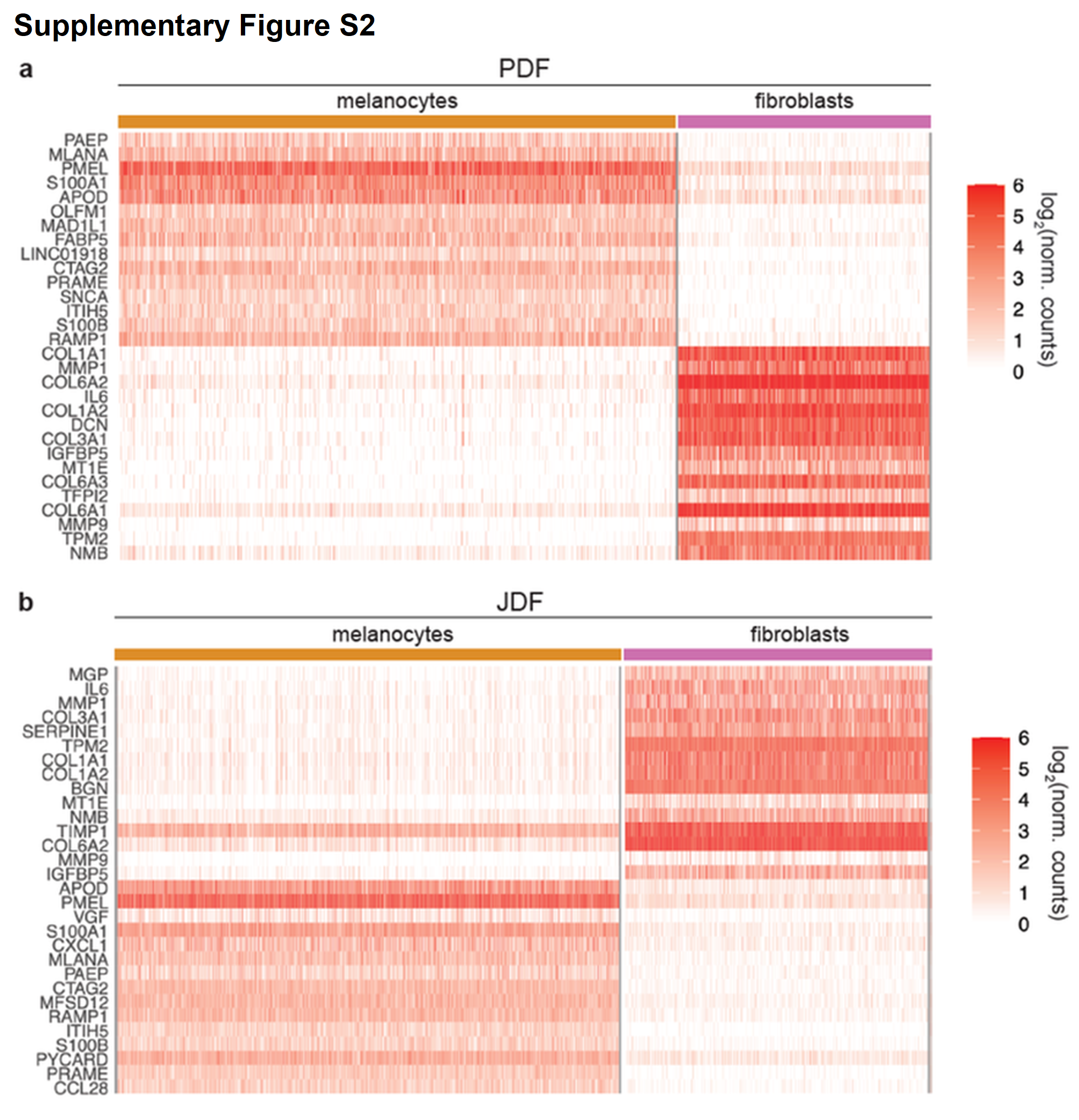

Supplement: Supplementary file 1 [file cancers-12-03324-s001.zip › Supplementary Figure S2.PNG]
